# Mid- to Long-Term Outcomes of Endovascular Aneurysm Repair Using the Ankura Stent Graft: An Eight-Year Single-Center Experience

**DOI:** 10.3390/medicina62071419

**Published:** 2026-07-22

**Authors:** Konstantinos Tigkiropoulos, Katerina Sidiropoulou, Georgios Chatziantoniou, Alexandros Apostolou, Kyriakos Stavridis, Christiana Anastasiadou, Dimitrios Karamanos, Nikolaos Saratzis

**Affiliations:** Division of Vascular Surgery, First Department of Surgery, School of Medicine, Faculty of Health Sciences, Aristotle University of Thessaloniki, Papageorgiou General Hospital, 56403 Thessaloniki, Greece; kasidi94@hotmail.com (K.S.); geochatziantoniou@gmail.com (G.C.); alexandrosgapo@gmail.com (A.A.); kstavridis17@yahoo.gr (K.S.); an.xristiana@hotmail.com (C.A.); dkaramanos@gmail.com (D.K.); nicos_saratzis@yahoo.com (N.S.)

**Keywords:** EVAR, Ankura stent graft, iliac limb occlusion, abdominal aortic aneurysm, aortic-related mortality

## Abstract

*Background and Objectives*: The Ankura stent graft was introduced in Europe in 2015 for the endovascular repair of infrarenal abdominal aortic aneurysms (AAAs). Evidence regarding its long-term efficacy remains limited. This study presents eight years of clinical experience with the Ankura endograft for endovascular aneurysm repair (EVAR) at a tertiary university vascular center. *Materials and Methods*: This single-center retrospective study included patients who underwent elective EVAR with the Ankura endograft between January 2015 and January 2023. All patients were anatomically suitable according to the instructions for use (IFU). Computed tomography angiography was performed at 1 and 12 months and annually thereafter during follow-up; the surveillance protocol was subsequently modified to use duplex ultrasound. Primary outcomes were categorized as early (technical success, clinical success, and 30-day mortality and morbidity) or late (aneurysm-related and non-aneurysm-related mortality). *Results*: A total of 220 patients were included (211 men [95.9%]); the mean age was 71.41 years (range, 49–87 years); and the mean aneurysm diameter was 59.15 mm (range, 32–109 mm). Primary and secondary technical success rates were 97.3% and 100%, respectively, and the clinical success rate was 96.8%. Thirty-day morbidity and mortality rates were 6.3% and 0%, respectively. No perioperative conversions occurred. The mean follow-up duration was 51.76 months (range, 1–131 months). Freedom from reintervention at 24, 36, 48, and 72 months was 98.2%, 97.7%, 97.2%, and 95.9%, respectively. Iliac limb patency was 99.5%. Aneurysm-related and non-aneurysm-related mortality during follow-up were 2.2% and 20.45%, respectively. *Conclusions*: In this study, the Ankura AAA stent graft demonstrated durable efficacy, with a high iliac limb patency rate, a low reintervention rate, and low aneurysm-related mortality during follow-up. Its long-term efficacy and safety should be evaluated in larger studies.

## 1. Introduction

The surgical management of aortic aneurysms has changed significantly over time, and endovascular aneurysm repair (EVAR) is currently considered the preferred treatment modality for patients with suitable anatomy [1]. Owing to its minimally invasive nature, EVAR is associated with lower 30-day perioperative mortality than open repair [2,3]. A variety of endografts are available in contemporary clinical practice, each with distinct technical characteristics designed to ensure adequate fixation, long-term device durability, conformability, and reduced mid- and long-term aneurysm-related mortality [4,5,6,7]. Several Asian-manufactured endografts have been introduced to the European market over the past decade, including Minos (MicroPort Endovascular MedTech Co., Shanghai, China) and Ankura (Lifetech Scientific, Shenzhen, China); their efficacy remains under investigation. Ankura is a relatively novel device that received CE marking in 2014. Its short-term efficacy and durability have been evaluated in a limited number of studies [8,9,10]. However, independent real-world data on its mid- and long-term performance remain limited. Therefore, this study aimed to present the eight-year clinical experience of a tertiary university vascular center and evaluate the mid- and long-term outcomes of EVAR using the Ankura endograft.

## 2. Methods

### 2.1. Study Design and Patient Population

All patients who underwent elective EVAR with the Ankura endograft for an infrarenal aortic aneurysm between January 2015 and January 2023 were retrospectively evaluated using a prospectively maintained database. Patients with ruptured aneurysms and juxtarenal aneurysms requiring adjunctive techniques (e.g., periscope or chimney techniques) were excluded. All patients were treated in accordance with the device instructions for use (IFU) and the European Society for Vascular Surgery (ESVS) guidelines [1]. In patients with saccular aneurysms, a lower diameter threshold (>30 mm) was applied in accordance with our institutional protocol. Patients with neoplastic disease were considered eligible for EVAR when their life expectancy exceeded 2 years, following consultation with the hospital’s oncologists. Preoperative planning using computed tomography angiography (CTA) was performed with RadiAnt DICOM Viewer (Medixant, Poznan, Poland) during the initial study period and with Endosize (Therenva, Rennes, France) after 2017. Endograft oversizing of 20% was applied on the basis of the nominal diameter of the proximal aortic neck. Our vascular center is part of one of the largest tertiary hospitals in northern Greece and performs 60–80 EVAR procedures annually. The surgical team comprises five senior vascular surgeons who are highly experienced in open and endovascular surgery and have worked together for the past 10 years. In total, 285 patients underwent elective EVAR during the study period; 220 received the Ankura endograft and were included in the analysis. The Ankura endograft was selected on the basis of its device characteristics (suprarenal fixation and an expanded polytetrafluoroethylene [ePTFE] graft material) and its routine on-site availability. All patients provided written informed consent before the procedure. The study received no external funding and was conducted in accordance with the Declaration of Helsinki. Approval was obtained from the hospital’s ethics/scientific committee (Papageorgiou General Hospital, 60-18/08/2025). Primary outcomes were categorized as early (technical success, clinical success, and 30-day morbidity and mortality) or late (aneurysm-related and non-aneurysm-related mortality). The characteristics of the Ankura AAA stent graft have been described previously and are not detailed further [8].

### 2.2. Procedural Technique

All procedures were performed in a hybrid operating room using a portable C-arm system (Ziehm Vision RFD, Nuremberg, Germany). General or regional anesthesia was used for all elective procedures. Bilateral femoral exposure was achieved through surgical cutdown in all cases, on the basis of operator preference and the unavailability of large-bore vascular closure devices at our center because of financial constraints. After systemic heparinization and complete deployment of the main body and iliac limbs, kissing-balloon angioplasty of the iliac limbs was routinely performed. Aortic ballooning was performed only when a type Ia endoleak was identified on final angiography. All patients were discharged on lifelong single-antiplatelet therapy or an antithrombotic regimen (warfarin or a direct oral anticoagulant) when indicated for a mechanical heart valve or atrial fibrillation.

#### 2.2.1. Follow-Up

According to our EVAR protocol, a follow-up visit at the outpatient clinic was scheduled for every patient treated in our department. If a patient was lost to follow-up, telephone contact was attempted to arrange a new appointment as soon as possible. Patients underwent computed tomography angiography (CTA) at 1 and 12 months after the procedure, followed by annual CTA surveillance for the first 4 years and every 2 years thereafter. During the final 4 years of the study period, the follow-up protocol was modified, and duplex ultrasound was introduced as the primary imaging modality after 12 months to reduce exposure to ionizing radiation and the risk of contrast-induced nephrotoxicity. When aneurysm sac enlargement was detected, CTA was subsequently performed to identify the underlying cause, including type Ia, Ib, II, or III endoleaks.

#### 2.2.2. Definitions

All definitions, including primary and secondary technical success, clinical success, cardiovascular events, and clinical outcomes, were based on the Society for Vascular Surgery reporting standards for patients undergoing EVAR [11]. Technical success was defined as the successful delivery and deployment of the Ankura stent graft without unintentional coverage of the visceral or iliac vessels and with no perioperative type I or III endoleak. Clinical success was defined as successful deployment of the endograft in the planned position, without aneurysm-related death, type I or III endoleak, graft migration (≥10 mm), stent graft thrombosis or infection, aneurysm rupture, or conversion to open repair. Perioperative morbidity and mortality were defined as complications and deaths occurring within 30 days of the procedure.

#### 2.2.3. Statistical Analysis

Statistical analyses were performed using SPSS for Windows (version 29; IBM Corp., Armonk, NY, USA). Continuous variables are presented as means and standard deviations (SDs). Categorical variables are presented as absolute numbers and percentages. Ninety-five percent confidence intervals (95% CIs) were calculated, and freedom from reintervention and overall survival were estimated using the Kaplan–Meier method. A *p* value < 0.05 was considered statistically significant.

## 3. Results

### 3.1. Baseline and Procedural Outcomes

Between 2015 and 2023, 220 patients were treated with the Ankura stent graft (211 men [95.9%]). Baseline clinical characteristics and demographics are presented in Table 1. The mean age was 71.4 years (range, 49–87 years; SD, 6.96; 95% CI, 71.4 ± 0.920), and the mean aneurysm diameter was 59.2 mm (range, 32–109 mm; SD, 12.55; 95% CI, 59.2 ± 1.658). The mean aortic neck diameter was 25.0 mm (range, 19–33 mm; SD, 3.31; 95% CI, 25 ± 0.437), the mean neck length was 19.2 mm (range, 15–31 mm; SD, 3.09; 95% CI, 19.3 ± 0.408), the mean infrarenal (β) neck angulation was 30° (range, 5–60°; SD, 9.29; 95% CI, 30 ± 1.228), the mean common iliac artery diameter was 15.4 mm (range, 9–24 mm; SD, 3.6; 95% CI, 15.4 ± 0.476), and the mean external iliac artery diameter was 8.1 mm (range, 7–12 mm; SD, 0.94; 95% CI, 8.1 ± 0.124) (Table 2). The mean endograft main-body diameter was 30.65 mm (SD, 3.39; 95% CI, 30.65 ± 0.448). The distribution of main-body diameters by device size is presented in Table 3. Saccular aneurysms were treated in 5 patients (2.3%). General anesthesia was used in 185 patients (84%), spinal anesthesia in 30 patients (13.6%), and local anesthesia with mild sedation in 5 patients (2.4%). Six type I endoleaks—three proximal (type Ia) and three distal (type Ib)—were identified on final angiography and successfully treated endovascularly with proximal cuff extension and iliac limb deployment, respectively. Type II endoleaks were observed in 20 patients (9%). Distal landing in the external iliac artery was not required in any case. Common femoral endarterectomy was required in 5 patients (2.3%) because of access-related calcified plaque rupture. One iliac limb occlusion occurred within 1 h postoperatively and presented as acute limb ischemia. The patient was successfully treated with Fogarty catheter thrombectomy, iliac limb relining, and common femoral endarterectomy. External iliac artery stenting was performed in 2 patients because of access-related dissection. No intraoperative conversion to open repair occurred. The mean operative time was 72.1 min (SD, 13.38), the mean fluoroscopy time was 7 min (SD, 2.85), and the mean contrast volume was 117.3 mL (SD, 32.9). Radiation dose data were unavailable for the first 54 patients because of lost records and were therefore excluded from the analysis. No patient was admitted to the intensive care unit, and the mean total hospital stay was 3.6 days (SD, 0.88). Thirteen patients developed contrast-induced nephropathy postoperatively, which was successfully managed with intravenous hydration. The 30-day morbidity and mortality rates were 6.3% and 0%, respectively (Table 4). Primary and secondary technical success rates were 97.3% and 100%, respectively, whereas the clinical success rate was 96.8%.

### 3.2. Follow-Up Outcomes

The mean follow-up duration was 51.8 months (range, 1–131 months; SD, 30.02; 95% CI, 51.76 ± 3.967). No patients were lost to follow-up. Three patients (1.3%) experienced aneurysm rupture caused by a type Ia endoleak at 12, 21, and 47 months. None had a type Ia endoleak on final angiography during the index procedure. Two patients underwent conversion to open repair with endograft explantation and aorto-aortic reconstruction using a tubular Dacron graft. Both died of hemorrhagic shock and multiorgan failure in the intensive care unit. Their most recent annual follow-up examinations, performed at our hospital 11 and 10 months before the index events, respectively, showed no evidence of endoleak or aneurysm enlargement. The third patient, who had completed 36 months of follow-up in our department, underwent urgent endovascular repair at another institution using a double-periscope technique and proximal cuff extension 1 month before his scheduled annual follow-up visit. He was discharged on postoperative day 5 in good condition.

During follow-up, type II endoleaks were observed in 25 patients (11.3%). The endoleak resolved spontaneously in 15 patients and remained stable without sac enlargement in 8; the remaining 2 patients developed aneurysm sac enlargement >10 mm. These patients underwent transperitoneal or translumbar embolization with coils and liquid embolic agents, performed by an interventional radiologist at our institution. Aneurysm size remained stable after the intervention throughout follow-up.

**Figure 1:**
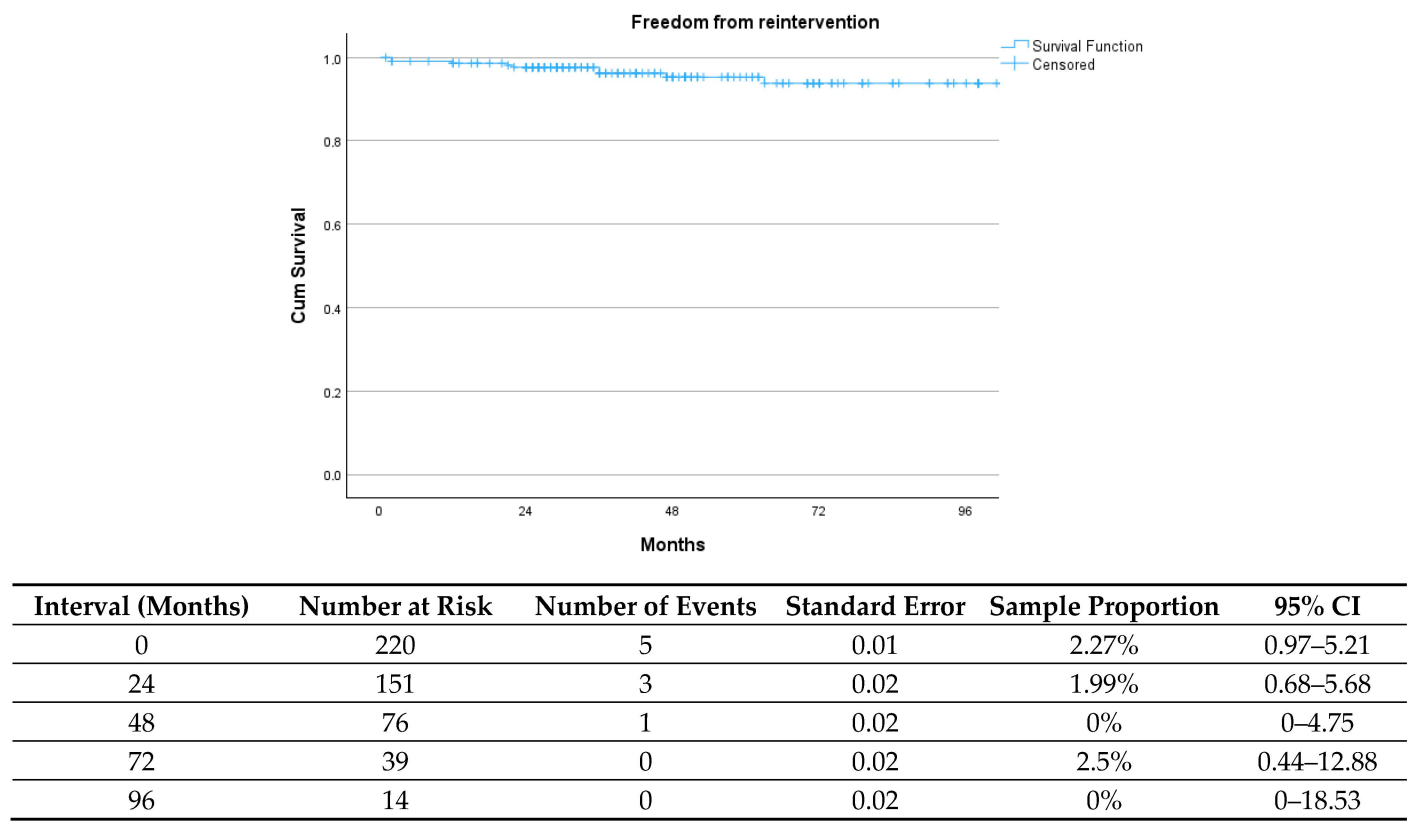
Kaplan–Meier estimate of freedom from reintervention during follow-up.

**Figure 2:**
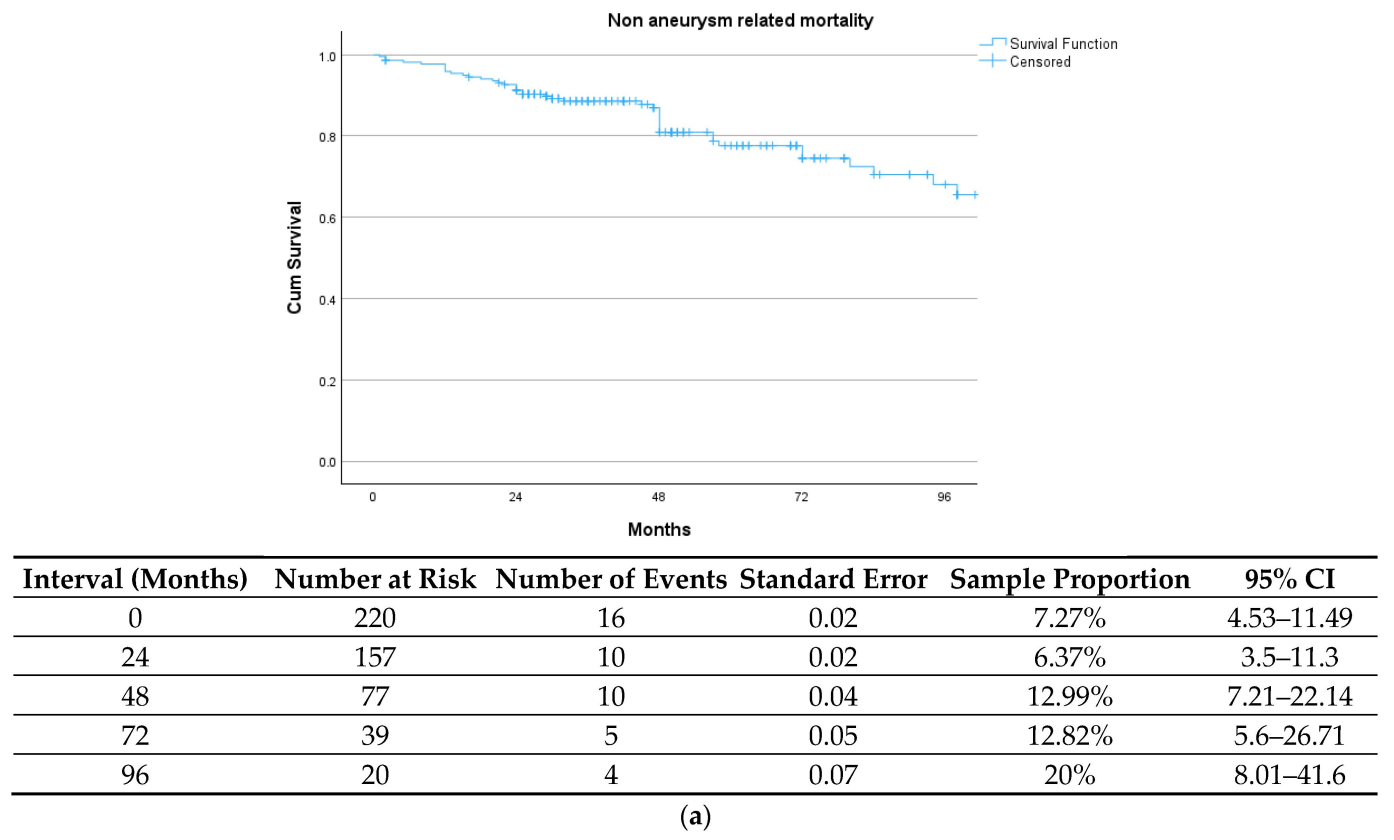

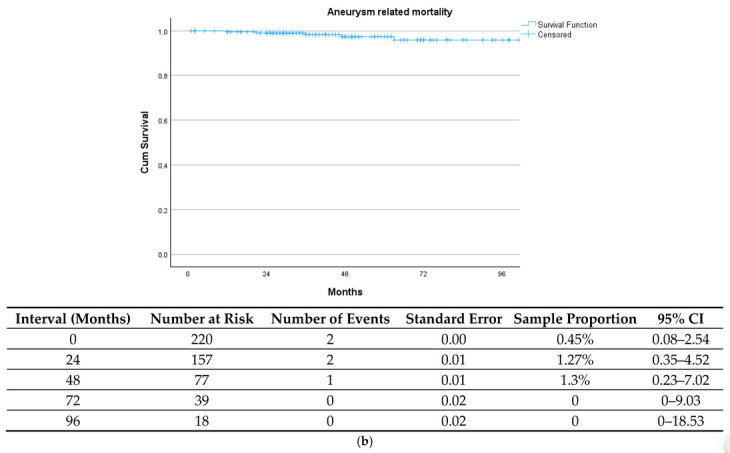
(**a**) Kaplan–Meier estimate of non-aneurysm-related survival. (**b**) Kaplan–Meier estimate of aneurysm-related survival.

One type IIIa endoleak was identified at 36 months. The patient presented with aneurysm rupture and hemorrhagic shock at another institution and underwent endovascular repair with iliac limb bridging; however, he died on the first postoperative day. No clear causative mechanism was identified. An aortoenteric fistula was diagnosed in one patient at 63 months. The patient presented with upper gastrointestinal bleeding and graft infection. He underwent axillobifemoral bypass, endograft explantation, and aortic stump ligation but died of multiorgan failure on postoperative day 10. No iliac limb thrombosis was observed during follow-up. Aortic-related mortality during follow-up was 2.2% (5 patients). Freedom from reintervention was 98.2% at 24 months, 97.7% at 36 months, 97.2% at 48 months, and 95.9% at 72 months. Non-aortic-related mortality during follow-up was 20.45% (45 patients). The most common cause was ischemic heart disease (n = 22), followed by malignancy (n = 12), COVID-19 infection (n = 7), and major stroke (n = 4). Kaplan–Meier curves for freedom from reintervention and aneurysm-related and non-aneurysm-related survival are presented in Figure 1 and Figure 2a,b.

## 4. Discussion

The present study represents one of the largest single-center European experiences evaluating the mid- and long-term performance of the Ankura stent graft since its introduction into clinical practice. In this cohort, EVAR with the Ankura endograft was associated with low aneurysm-related mortality (2.2%), excellent iliac limb patency, and high freedom from reintervention (95.9% at 72 months).

The 12-month efficacy of the Ankura stent graft has been evaluated in a limited number of studies [8,9,10]. Across a total of 268 patients, primary and secondary technical success rates were 97% and 100%, respectively. Freedom from reintervention was 98.5%. No perioperative conversions occurred. Aneurysm-related mortality was 0%, and iliac limb patency was 99.6%. A recent study by Akca Ozsar et al. [12] evaluated the mid-term outcomes of EVAR using the Ankura endograft in 100 patients in a real-world postmarket follow-up study. Technical success was achieved in all patients, with major adverse events occurring in 2% of the study population at 30 days (one myocardial infarction and one acute kidney injury). However, during the 12- to 24-month follow-up period, a substantial number of type Ib endoleaks were detected (n = 23), resulting in numerous reinterventions with iliac extensions to achieve distal aneurysm sealing. The reintervention rate was considerably higher than that reported in contemporary EVAR studies. The authors stated that progression of aneurysmal disease in the iliac arteries was the main etiological factor; however, suboptimal iliac oversizing during the index procedure may also have contributed. They did not report the degree of oversizing, an important parameter when evaluating stent graft efficacy during follow-up. Their findings contrast with those of the present study, in which endograft oversizing was 20% in all patients and no type Ib endoleaks occurred during follow-up.

Although EVAR is currently considered the preferred treatment modality for infrarenal AAA according to the ESVS guidelines [1], concerns regarding long-term durability remain, and data extending beyond mid-term follow-up are relatively limited [6,7,13,14].

The durability of EVAR depends not only on device integrity, which has improved with newer-generation endografts, but also on the progression of aneurysmal disease, particularly at the proximal and distal sealing zones. The Ankura stent graft is a bimodular endograft composed of a bilayer ePTFE membrane covering supported by nitinol stents, providing low permeability and biocompatibility [8]. The main-body delivery system ranges from 20 to 22 Fr. A connecting bar in the final two waves of the main body and in the limb prevents graft shortening and provides flexibility. The stents are enclosed between the ePTFE membranes and thermally bonded to them, minimizing pinhole leakage.

Aortic neck dilatation (AND) is a key mechanism of late failure after EVAR. In a systematic review by Chatzelas et al. [15], the pooled incidence of AND was 22.9%, and AND was associated with a significantly increased risk of type Ia endoleak. Similarly, long-term geometric remodeling of the proximal aortic neck has been reported across different endografts, with clinically significant dilatation observed in a substantial proportion of patients over time [16]. Malach et al. [17] further demonstrated that suprarenal fixation may influence changes in aortic diameter during follow-up. Oliveira et al. [18] investigated AND in a large retrospective single-cohort study. They examined postoperative CT scans obtained after a median follow-up of 3.3 years in 460 patients. Overall, 8.5% of patients had >20% dilatation by the end of follow-up; the median dilatation was 11.1%, and the greatest increase occurred during the first postoperative year. The pathophysiology of AND remains unclear; however, inflammatory aortic remodeling induced by aortic stiffness was proposed by Schellinger et al. [19] in an ex vivo experimental study using porcine aortic models. Endograft implantation increased aortic stiffness in the stented aortic segment, and a significant stiffness gradient was also observed at the interface between the stented and unstented aorta, corresponding to the level of the aortic neck. In the present study, three patients developed a type Ia endoleak associated with aortic neck dilatation at 12, 21, and 47 months, respectively, ultimately leading to aneurysm rupture. Two patients died after conversion to open repair, whereas one was successfully treated endovascularly using proximal cuff extension and chimney techniques. These findings highlight the importance of long-term surveillance and the role of disease progression in late EVAR failure.

Postoperative imaging surveillance is mandatory after EVAR to detect or predict potential complications. CTA is considered the gold-standard examination; however, the cumulative radiation-associated cancer risk is an important consideration, particularly in younger patients. Duplex ultrasound, especially contrast-enhanced ultrasound (CEUS), is an alternative imaging modality that can detect endoleaks and measure aneurysm diameter at low cost and without ionizing radiation or contrast-induced nephrotoxicity. In a meta-analysis, the pooled sensitivity of CEUS was 0.98 (95% CI, 0.90–0.99; I^2^ = 0.32), and the pooled specificity was 0.88 (95% CI, 0.78–0.94; I^2^ = 0.67) [20]. The diagnostic accuracy of CEUS for detecting endoleaks after EVAR was also established in a systematic review and meta-analysis by Kapetanios et al. [21], in which the pooled sensitivity and specificity for all endoleaks were 0.94 (95% CI, 0.89–0.97) and 0.93 (95% CI, 0.89–0.96), respectively. In our study, modification of the follow-up protocol did not affect the detection of adverse events during the surveillance period.

Iliac limb occlusion is a recognized complication following EVAR, with clinical manifestations ranging from intermittent claudication to acute limb ischemia. In a meta-analysis including 5454 patients, the reported rate of limb occlusion was 5.6% [22]. Several mechanisms have been implicated, including excessive graft oversizing, limb kinking, and extension into the external iliac artery [23,24]. In the present cohort, only one patient developed iliac limb thrombosis in the perioperative period, likely due to poor distal outflow, and was successfully managed with a hybrid approach. Notably, no cases of late limb occlusion were observed during follow-up. This may be related to procedural factors, such as routine kissing balloon molding, as well as device characteristics; however, this observation remains speculative.

Despite the well-established perioperative benefits of EVAR over open repair, durability beyond 5 years remains a key concern. Long-term outcome data are limited, and most available evidence derives from industry-sponsored registries [6,7]. In the ENGAGE registry, 5-year aneurysm-related survival was 97.8%, freedom from rupture was 98.6%, and overall survival was 67.4% [25]. Similarly, the GREAT registry reported 5-year freedom from aortic-related mortality of 98.8%, a reintervention rate of 7.2%, and overall survival of 71.2% for the Gore Excluder device [26]. Spanos et al. [14] presented the long-term outcomes of patients treated electively with the Endurant endograft (Medtronic, Santa Rosa, CA, USA). The study included 361 patients. The 30-day mortality rate was 0.6%, and the major complication rate was 4.1%. Survival was 81% (SE, 4.8%), 72% (SE, 6.4%), and 52% (SE, 9.2%) at 4, 6, and 8 years, respectively, and aneurysm-related mortality was 1.7%. In the present study, the Ankura endograft demonstrated comparable performance, with sustained durability reflected in high freedom from reintervention and low aneurysm-related mortality.

In addition to Ankura, other Asian-manufactured endografts have been introduced to the European market over the past decade. One such novel stent graft is Minos (MicroPort Endovascular MedTech Co., Shanghai, China), a low-profile (14–16 Fr) polyester device with suprarenal fixation that received CE marking in 2019. Its efficacy has been evaluated in a limited number of European studies, with high clinical success rates reported during 12 months of follow-up [27,28]. However, these results are preliminary, and further studies are required to compare the efficacy of both endografts over the mid- and long-term.

This study has several limitations. It was a single-center retrospective analysis with a relatively limited sample size, although it reflects real-world, non-sponsored clinical practice. In addition, the study population was treated according to the IFU criteria, which may explain the high freedom from reintervention during follow-up. The population was also almost entirely male, with only 9 women; both factors should be acknowledged as relevant limitations. Women undergoing EVAR have different anatomical characteristics, rates of access-related complications, and perioperative outcomes. A systematic review by Marzano et al. examining sex differences in outcomes after EVAR reported an odds ratio (OR) of 1.73 (95% CI, 1.32–2.26) for 30-day mortality and an OR of 1.90 (95% CI, 1.43–2.53) for in-hospital mortality in women compared with men. Women also had increased risks of limb ischemia (approximately 2.4-fold), renal complications (OR, approximately 1.7), and cardiac complications (OR, approximately 1.7), as well as higher long-term all-cause mortality (hazard ratio, 1.23; 95% CI, 1.09–1.38) [29]. Another limitation was that aneurysm-related and non-aneurysm-related mortality were analyzed using separate Kaplan–Meier curves, despite the relatively high non-aneurysm-related mortality. Consequently, these competing events may have biased the estimation of aneurysm-related mortality. However, the absolute number of aneurysm-related deaths was low, and dividing the cohort into smaller independent groups would have produced extremely wide confidence intervals and less meaningful conclusions. Nevertheless, this study provides valuable mid- to long-term outcome data over an 8-year period and represents one of the first independent European experiences evaluating the Ankura stent graft.

## 5. Conclusions

In a real-world setting, EVAR using the Ankura stent graft is associated with high technical success, low aneurysm-related mortality, and sustained durability during mid- to long-term follow-up. These findings support its use as a reliable endovascular option for the elective treatment of infrarenal AAA. Further large-scale prospective studies are warranted to confirm long-term outcomes.

## Figures and Tables

**Table 1:** Baseline characteristics and demographics of the study population.

Study population	220 Patients; 211 Men (95.9%)
Age	71.4 years (mean), 95% CI 71.4 ± 0.920
Hypertension	184 pts (83.6%)
Ischemic heart disease	138 pts (62.7%)
Dyslipidemia	143 pts (65%)
COPD	29 pts (13.1%)
Prostatic hypertrophy	46 pts (21.8%)
Atrial fibrillation	28 pts (12.7%)
Diabetes mellitus	32 pts (14.5%)
Kidney disease	19 pts (8.6%)
Previous or current smoking	185 pts (84%)
Preoperative creatinine	Mean, 1.22 mg/dL; 95% CI, 1.22 ± 0.0433
Body mass index	Mean, 20.22; 95% CI, 20.22 ± 0.481

**Table 2:** Anatomical characteristics of abdominal aortic aneurysms in the study population.

Fusiform AAA	215 pts (97.7%)
Mean aneurysm diameter	Mean, 59.15 mm (range, 32–109 mm); 95% CI, 59.2 ± 1.658
Mean aortic neck diameter	Mean, 25.02 mm (range, 19–32 mm); 95% CI, 25 ± 0.437
Mean aortic neck length	Mean, 19 mm (range, 15–31 mm); 95% CI, 19.3 ± 0.408
Mean β neck angle	30° (range, 5–60°); 95% CI, 30 ± 1.228
Mean common iliac artery diameter	Mean, 15.4 mm (range, 9–24 mm); 95% CI, 15.4 ± 0.476
Mean external iliac artery diameter	Mean, 8.1 mm (range, 7–12 mm); 95% CI, 8.1 ± 0.124

**Table 3:** Distribution of main-body diameters by device size.

Main Body Graft Size (mm)	Number of Patients (220, 100%)
24	5 (2.5%)
26	25 (11.3%)
28	59 (26.8%)
30	32 (14.5%)
32	35 (15.9%)
34	32 (14.5%)
36	32 (14.5%)

**Table 4:** Thirty-day postoperative morbidity during the study period.

Postoperative Morbidity	Total Number of Patients (14, 6.3%)
Contrast-induced nephrotoxicity	13 patients (5.9%)
Iliac limb occlusion	1 patient (0.4%)

## Data Availability

The original contributions presented in this study are included in the article. Further inquiries can be directed to the corresponding author.
